# Monitoring and modulating interconnected physiological systems in space using portable closed-loop technologies

**DOI:** 10.3389/fnetp.2026.1867627

**Published:** 2026-07-30

**Authors:** Enrico De Martino, Peng Lyu, Ikram Brahim, Mehaboobathunnisa Sahul Hameed, Lars Arendt-Nielsen, Yacine Hadjiat

**Affiliations:** 1 Center for Neuroplasticity and Pain (CNAP), Department of Health and Technology, Aalborg University, Aalborg, Denmark; 2 Dubai Health Innovations, Mohammed Bin Rashid University of Medicine and Health Sciences, Dubai, United Arab Emirates

**Keywords:** biopsychosocial pain, multisystem dysregulation, network physiology, neuromodulation, sleep disturbance, spaceflight physiology, stress regulation, wearable sensors

## Abstract

Human spaceflight exposes individuals to prolonged, multifactorial stressors, including microgravity, radiation, isolation, confinement, altered light-dark cycles, and operational demands, that affect biological, cognitive, psychological, behavioral, and social domains. These stressors may contribute to body deconditioning, dysregulated stress responses, sleep disruption, increased pain vulnerability, impaired cognition, mood disturbances, and interpersonal conflict. As human space exploration moves toward longer missions beyond low Earth orbit, effective countermeasures will require small, portable, autonomous, low-power technologies capable of continuously monitoring interconnected systems and delivering personalized interventions in real-time. In this perspective, we propose that the consequences of spaceflight are best understood through a network physiology framework, in which stress regulation, sleep, pain, cognition, mood, social interaction, and other physiological functions are viewed as dynamically coupled components of an integrated system. Within this framework, disturbances in one domain may propagate to other domains, reducing resilience and increasing vulnerability to multisystem dysfunction. We discuss how advances in multimodal wearable and habitat-integrated sensing, combined with AI-based analysis, enable continuous monitoring in space-relevant environments. Body-worn and ambient sensors can capture neural, autonomic, cardiovascular, thermal, and behavioral signals, enabling longitudinal assessment of system-level adaptation. Integrating these signals with AI-based analysis may help identify deviations from adaptive network states, derive markers of multisystem resilience, and guide personalized countermeasures. We further discuss the potential of AI-guided, closed-loop, non-pharmacological interventions to restore physiological balance and maintain performance during long-duration missions. Beyond spaceflight, this framework may also inform precision health approaches to multisystem dysfunction on Earth.

## Multidomain consequences of spaceflight stressors

Human space exploration is entering a new era, referred to as the ‘Second Space Age” (Mason et al., 2024), characterized by plans for long-duration missions beyond low Earth orbit. NASA and the European Space Agency (ESA) are developing the Artemis program, which aims to establish a sustained human presence on the Moon (NASA, 2020). Similarly, the China National Space Administration is planning to land astronauts on the Moon before 2030 (Chunlai et al., 2019). Roscosmos is developing a new Russian Orbital Station to maintain a long-term national orbital presence following the International Space Station program (Grigor’ev et al., 2021). Furthermore, several private companies are building commercial stations for stays in low Earth orbit (Zea et al., 2024). Collectively, these initiatives point toward the future of sustained human presence in space.

**FIGURE 1:**
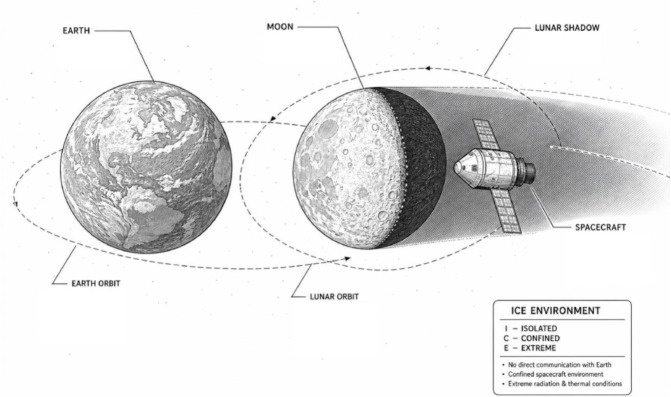
The isolated, confined and extreme (ICE) environment of deep-space exploration. A spacecraft can face communication blackouts, spatial confinement and thermal stressor conditions while transiting between Earth and lunar orbit. An example of a challenge to multisystem physiological regulation.

During space missions, astronauts are exposed to multiple space-related stressors. These include extreme conditions such as microgravity, cosmic radiation, and altered light-dark cycles (Willey et al., 2021). Astronauts also live in protected habitats that introduce additional stressors, including limited resources such as food and power, elevated carbon dioxide concentrations, poor ventilation, and confinement (Scully et al., 2019). These conditions limit privacy, require shared living spaces, and may substantially reduce contact with family and friends (Yin et al., 2023). Work in space is often characterized by alternating periods of intense workload and relative inactivity, which may further increase psychological and physiological stress (Jones et al., 2022). Together, these factors create a complex stress environment with consequences across biological, cognitive, psychological, behavioral, and social domains, whose interactions may promote maladaptive responses (Figure 1).

Among space-related stressors, microgravity is the most distinctive and physiologically impactful, inducing body deconditioning with effects across multiple body systems (Demontis et al., 2017). For instance, fluid shifts toward the upper body and head lead to cardiovascular and cerebrovascular adaptations, including changes in plasma volume and vascular remodeling (Alzaabi et al., 2025). Microgravity also affects musculoskeletal integrity, causing bone loss, muscle atrophy, and altered neuromuscular control (Lee et al., 2022; Man et al., 2022). Exercise remains the principal countermeasure during spaceflight, but its protective effects are incomplete, and deconditioning persists across multiple systems (Scott et al., 2023). Microgravity also disrupts sensorimotor integration by altering vestibular, proprioceptive, and visual inputs, thereby impairing balance, spatial orientation, and coordinated movement (Tays et al., 2021). Astronauts may also develop ocular alterations collectively referred to as spaceflight-associated neuro-ocular syndrome, which involves structural eye changes and visual disturbances (Lee et al., 2020). Beyond the direct biological effects of microgravity, combined exposure to habitat-related, operational, and psychosocial stressors places sustained demands on stress-regulation systems (Capri et al., 2025). Studies have reported changes in autonomic regulation, including increased sympathetic activity, suggesting alterations in neuroautonomic control during space missions (Blaber et al., 2004). Changes in endocrine stress markers, such as cortisol, have also been observed before, during, and after spaceflight (Macho et al., 2003).

Stress processing begins in the nervous system, where environmental demands are evaluated (Lazarus, 1993). These signals activate the sympathetic nervous system and the hypothalamic–pituitary–adrenal axis, leading to the release of catecholamines and glucocorticoids that support adaptation to environmental challenges (Myers et al., 2014; Herman et al., 2016). Clinically, stress responses manifest as increased heart rate, reduced heart rate variability, altered respiratory patterns, and shifts in peripheral thermoregulation (Kim et al., 2018). Behaviorally, stress-related dysregulation often emerges as disturbed sleep, reduced sleep efficiency, and circadian misalignment (Lo Martire et al., 2020). Furthermore, altered lighting, confinement, and noise can disrupt circadian rhythms, leading to sleep fragmentation and reduced sleep efficiency (Zong et al., 2025). Stress-regulation systems are closely intertwined with sleep physiology, and stress dysregulation can impair both sleep quality and circadian timing. Sleep disturbance may further amplify stress reactivity, as altered light-dark cycles, confinement, and workload frequently disrupt sleep (Yap et al., 2020). This bidirectional interaction may represent a key pathway through which dysfunction propagates across multiple domains. Its operational importance is underscored by the frequent use of pharmacological support during spaceflight, especially sleep-promoting medications (Wotring, 2015). Although such medications may provide short-term symptom relief, they do not address the interacting physiological and behavioral mechanisms that underlie dysfunction and its propagation across domains.

Stress-related dysregulation may also impair cognitive, psychological, and social functioning. Cognitive effects may include reduced concentration and impaired executive function (Shields et al., 2016). Astronauts often report mental fatigue, difficulty concentrating, and a reduced ability to sustain high levels of cognitive performance, a cluster of symptoms often referred to as “space fog” (Kuldavletova et al., 2023). Psychological dysregulation may manifest as mood disturbance, increased anxiety, irritability, and reduced motivation (Mariotti, 2015) and psychiatric symptoms, including depression and irritability, have occurred during orbital spaceflight (Friedman and Bui, 2017). At the social level, stress-related dysregulation may impair interpersonal functioning by increasing irritability and reducing communication quality, effects that may be further exacerbated by social isolation, confinement, limited privacy, interpersonal tension, and reduced social support, with reductions in team cohesion also reported during space missions (Vinokhodova et al., 2023). To mitigate stress-related dysregulation, mindfulness has been proposed as a strategy to support cognitive, psychological, and social wellbeing (Pagnini et al., 2019), in addition to medication use (Wotring, 2015).

Stress-regulation systems are closely connected to pain-processing mechanisms, suggesting that alterations in stress physiology during spaceflight may influence pain perception. These interactions may contribute to increased pain sensitivity and muscle tension, which are commonly observed under prolonged stress (Shanthanna et al., 2022). Headaches and low back pain are frequently reported during space missions (Ceniza-Bordallo et al., 2024; van Oosterhout et al., 2024), and analgesics are among the pharmacological agents used during spaceflight, underscoring the operational importance of pain management during orbital and long-duration missions (Wotring, 2015). These symptoms may reflect biological adaptations to microgravity, such as fluid shifts and spinal elongation, as well as cumulative stress-related behavioral and emotional factors. Pain is a multidimensional experience shaped by sensory, emotional, cognitive, and social processes (Raja et al., 2020). In many pain conditions, the relationship between pain and structural tissue abnormalities is weak, suggesting that pain perception is strongly influenced by neural mechanisms and the balance between facilitatory and inhibitory processes (Arendt-Nielsen et al., 2015; Maher et al., 2017). Clinical evidence indicates that psychological and behavioral factors, including stress, sleep disturbance, negative affect, and cognitive load, can increase pain vulnerability through shared neural circuitry (Gerhart et al., 2018). In extreme operational environments such as spaceflight, where sleep disruption, confinement, cognitive strain, and social stress coexist, pain may therefore emerge as an integrated manifestation of multisystem dysregulation rather than as an isolated symptom.

**FIGURE 2:**
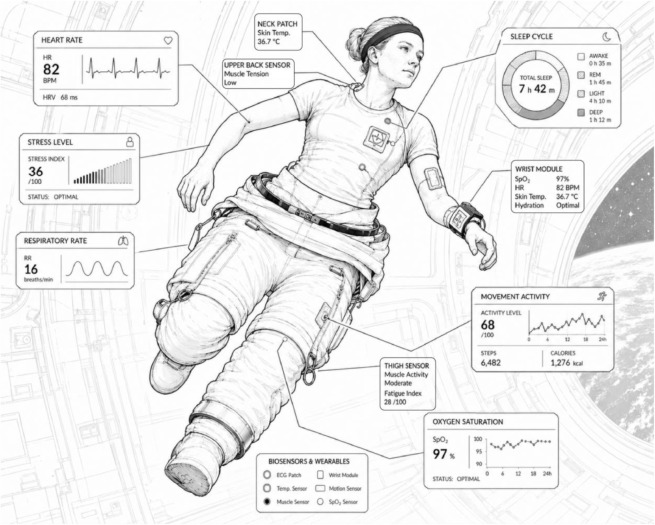
Multimodal wearable sensing system for continuous astronaut health monitoring via biosensors and wearables that capture physiological signals.

**FIGURE 3:**
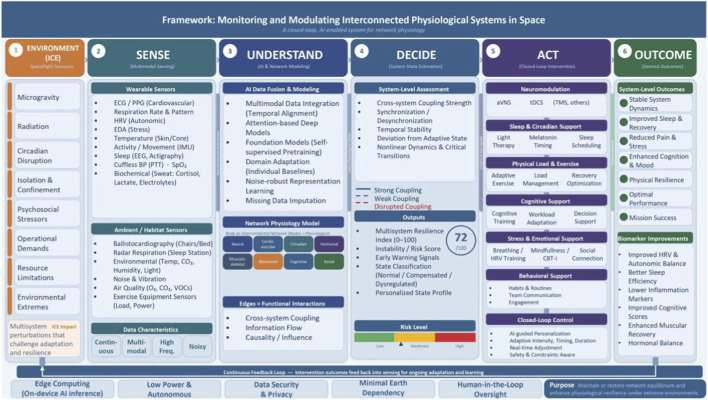
Network physiology framework for closed-loop health monitoring and intervention in isolated, confined, and extreme environments.

Taken together, these findings suggest that the effects of spaceflight should not be seen as isolated changes but as interconnected parts of a larger adaptive system. Body deconditioning, stress regulation, sleep, pain, cognition, mood, and social interaction are strongly linked, meaning issues in one area can spread to others and push the organism from adaptive regulation into multisystem dysfunction. Using this network physiology approach, understanding how these systems interact could help find early signs of vulnerability and develop targeted countermeasures for long-duration missions (Figure 2). Although previous work has examined multimodal monitoring, AI-based decision support, and closed-loop neuromodulation separately, we propose an integrated model in which coupling metrics from network physiology serve as the primary means of assessing system status and steering adaptive, multidomain, non-pharmacological interventions during extended space travel (Figure 3).

## Monitoring interconnected systems in constrained environments

Building on the view that astronaut health reflects dynamic interactions across biological, cognitive, psychological, behavioral, and social domains, the next challenge is to determine how to monitor these interactions in operational spaceflight settings. Rather than treating physiological variables as isolated targets, the framework proposed here emphasizes network-level coupling and decoupling patterns as the key state variables for detecting early deviations from adaptive regulation. Such monitoring requires technologies capable of detecting early multisystem changes while remaining compatible with the space, power, and autonomy constraints of long-duration missions. Long-term astronaut health monitoring depends on systems that are small, low-power, minimally obtrusive, and capable of autonomous operation over extended periods. On space stations, where personal space is highly constrained, sensing technologies must balance clinically relevant measurement objectives against the operational and physical limitations of onboard hardware (Blaber et al., 2004; Scully et al., 2019; Jones et al., 2022). Recent advances in multimodal sensing, AI-based physiological analysis, and network-based modeling provide a promising basis for meeting this need.

Among these advances, AI-based physiological modeling has emerged as a powerful framework for multimodal data fusion (Li and Zhang, 2025), integrating signals such as electrocardiography, photoplethysmography, respiration, accelerometry, and electrodermal activity to characterize interactions among physiological systems, complementing rather than replacing isolated biomarker monitoring. These models use complementary temporal and frequency-domain information from different data sources, thereby improving robustness to noise and variable signal quality, particularly in ambulatory settings. Attention-based models build on this concept by assigning dynamic weights to time windows and channels that are most physiologically informative for detecting subtle changes in autonomic tone, microarousals, or stress-related sympathetic activation (Park et al., 2022). More recently, physiological foundation models have shown that self-supervised pretraining on large-scale physiological datasets can capture generalizable latent regulatory dynamics, enabling prediction with relatively few labeled data and supporting personalization across individuals (Thapa et al., 2026). These AI-based approaches have also been applied to sleep stage detection (Morokuma et al., 2023), mental stress detection (Ghosh et al., 2022), cognitive-load detection (Singh et al., 2026), and acute pain assessment (Gkikas and Tsiknakis, 2023). In these applications, neural networks have identified subtle shifts away from adaptive regulation before apparent symptoms emerge. The proposed framework draws on established methods from network physiology and computational neuroscience. Cross-correlation and spectral coherence analysis quantify linear coupling strength and frequency-specific synchrony between physiological time series, for example, between heart rate variability and respiratory rate (Mohamed et al., 2018; Terry et al., 2016), or between slow-wave sleep oscillations and autonomic tone (Mohsen et al., 2019), providing a multivariate profile of dynamic intersystem coordination. Furthermore, graph-based modelling (Yildiz and Subasi, 2026) may be used to represent and track dynamic information flow between physiological signals, enabling quantification of directional information flow between nodes, and giving insight into which physiological signal is driving the current state at a given time point. Some of these methods will require not only adaptation and validation, but systematic assessment given the unique challenges of spaceflight environments. Transfer entropy analysis may further be applied to quantify directional information flow between physiological nodes, enabling identification of which subsystem is driving network disruption at a given time point.

Within a network physiology framework, the multimodal signals captured through physiological AI represent coupled neural, autonomic, cardiovascular, circadian, and behavioral nodes of an integrated system (Bashan et al., 2012; Hirvonen et al., 2018). Instead of monitoring individual biomarkers in isolation, it may be critical to examine patterns of dynamic cross-system coupling and decoupling and to identify early deviations from adaptive network states. Although direct validation of network-level physiological metrics in human spaceflight populations remains limited, evidence from terrestrial network physiology, sleep-stress interaction research, and multidomain systems modelling suggests that patterns of cross-system coupling may capture early physiological state transitions that are not readily detectable using isolated biomarkers alone (Bashan et al., 2012).

For example, a combination of declines in heart rate variability, previously reported during spaceflight in association with autonomic shifts (Blaber et al., 2004), alterations in slow-wave sleep (Jones et al., 2022), and peripheral body temperature changes may portend a stress-related physiological shift well in advance of psychological or cognitive symptoms. Similarly, patterns of muscle activation, together with sleep fragmentation and autonomic imbalance, may indicate increased vulnerability to stress-related pain augmentation, given overlapping circuitry in stress and pain neural processing (Shanthanna et al., 2022; Gerhart et al., 2018).To obtain a more complete picture of this physiological network, the monitoring system should also capture interactions among electrical activity, blood flow, and hormonal control. Pulse transit time (PPT) and photoplethysmography (PPG)-derived cuffless blood pressure provide information about cardiovascular dynamics shaped by blood volume and vascular compliance (Mukkamala et al., 2015). Within a network framework, these parameters are not merely indicators of fluid shifts. They also function as nodes reflecting the mechanical and autonomic coupling between the heart and vasculature (Mukkamala et al., 2015). This systemic map may be further enriched by emerging wearable biochemical sensors, including sweat-based cortisol detection and interstitial fluid biosensors (Gao et al., 2016; Heikenfeld et al., 2018). Together, these electrical, chemical, and hemodynamic signals may allow monitoring systems to capture a broader network of homeostatic functions under extreme environmental conditions.

Within the monitoring framework, radiation exposure is treated as an upstream environmental perturbation capable of influencing multiple interconnected physiological systems dynamics. While direct biological dosimetry remains technically challenging during long-duration missions, radiation-related effects can be inferred through downstream biomarkers—including inflammatory signaling, oxidative stress, autonomic imbalance, sleep disruption, and cognitive changes. AI models integrate these signals with exposure estimates to enable individualized risk stratification and early detection of radiation-induced dysfunction.

AI and machine learning (ML) methods play an essential role in extracting clinically relevant information from multimodal data. Multimodal fusion, attention-based neural networks, and foundation models have shown the ability to identify hidden physiological representations that reflect the holistic state of the physiological system rather than individual signal manifestations (Moor et al., 2023). For deep-space expeditions with limited astronaut-earth communication, these models can be designed to adhere to the principles of edge computing, i.e., execute on the device itself, perform adaptive sampling, and use efficient architectures (Lane et al., 2016). Modulating the sampling frequency in response to potential physiological instability can save significant power while remaining sensitive to the early manifestations of dysfunction. Importantly, space-compatible monitoring should aim to produce interpretable indices of multisystem resilience rather than large volumes of raw physiological data. Composite markers that indicate cross-system coupling strength and network stability can provide useful measures of physiological robustness for crew members and mission operators alike. Such indices could enhance risk stratification of sleep disruption, cognitive fatigue, autonomic instability, and stress-related pain vulnerability during prolonged missions by enabling early detection of deviations from personalized adaptive states.

Building on this, the next step is to extract latent physiological representations and to turn these representations into stable and useful system-level metrics. In network physiology, this means projecting high-dimensional model outputs onto low-dimensional indices that retain information about cross-system coupling, temporal stability, and adaptive capacity. Such representations remain stable despite changes in the environment or sensor, while still being sensitive to deviations from an individual’s baseline dynamics. This enables an efficient decision support for monitoring physiological resilience without constant ground-based analysis.

To further reduce the burden of individual sensors, habitat-integrated sensing can be employed. Embedded ballistocardiography sensors in chair components, radar sensors to measure respiratory rate in the sleep station, and exercise hardware with integrated sensors have all been demonstrated in terrestrial and analog settings (Etemadi and Inan, 2018; Hassanpour and Yang, 2025). This suite of sensors could be deployed in a small habitat with limited personnel to monitor physiological parameters passively as part of the crewmembers’ daily routines. Combined with minimal-sensor architectures, garment and habitat integration, and AI-driven multimodal analysis, space-monitoring systems can move from episodic measurement to continuous, low-burden assessment of interconnected physiological regulation. This approach is both technologically feasible and clinically applicable, enabling proactive system-level health management during long-duration exploration missions.

## Modulating interconnected systems in constrained environments

Future countermeasures for multisystem alterations arising from prolonged exposure to microgravity, confinement, and other mission-related stressors will require strategies that actively regulate interconnected processes across biological, cognitive, psychological, behavioral, and social domains. Within this framework, the goal is to restore adaptive network dynamics across interacting domains. By continuously analyzing multimodal physiological data, these systems may detect early deviations from adaptive network states and deliver targeted interventions before apparent dysfunction emerges. In this context, AI can support the integration and interpretation of multimodal signals, thereby enabling the detection of early deviations from adaptive states. Closed-loop frameworks can then use this information to continuously adjust interventions in real-time based on the astronaut’s current condition. Closed-loop systems receive continuous streams of multimodal data from wearable and habitat-integrated sensors, including neural, autonomic, cardiovascular, circadian, and behavioral signals from which clinically relevant biomarkers are extracted, such as heart rate variability, sleep architecture, respiration, activity level, and indices of thermoregulation. Instead of single parameters, closed loop systems use composite and cross-domain biomarkers that reflect the dynamic coupling between physiological systems. Changes in autonomic balance, sleep fragmentation, and circadian misalignment may indicate early dysregulation, while changes in activity, muscle activity, and cardiovascular response might signal an early deconditioning or fatigue. By constantly monitoring these biomarker profiles, closed-loop systems can tailor intervention strategies depending on the current network state of the individual’s neural, behavioral, and physiological processes. AI-guided closed-loop approaches may therefore support interventions across multiple domains. For example, such approaches could modulate neural and autonomic function through neuromodulation, adjust light exposure and sleep timing to stabilize circadian rhythms, adapt physical workload to mitigate deconditioning, provide cognitive support during periods of fatigue or high demand, deliver personalized psychological interventions for stress and mood disturbance, and support social functioning by identifying early signs of interpersonal strain and promoting adaptive communication. Among these approaches, non-invasive neuromodulation offers a means of modulating neural and autonomic pathways involved in sleep regulation, emotional regulation, and cognitive control and closed-loop control (Zrenner and Ziemann, 2024), these approaches may enable personalized, adaptive interventions that respond to ongoing physiological and behavioral changes rather than relying on fixed stimulation protocols. For instance, auricular vagus nerve stimulation (aVNS) targets afferent branches of the vagus nerve and can modulate central autonomic networks involved in stress regulation, inflammation, and pain processing (Bu et al., 2026). When integrated into closed-loop systems, stimulation parameters such as intensity, frequency, and timing can be dynamically adjusted based on real-time physiological signals, including heart rate variability, sleep architecture, and indicators of autonomic tone.

Importantly, chronic radiation exposure and microgravity-induced physiological adaptations may alter neural excitability, autonomic responsiveness, vascular function, and tissue sensitivity, thereby influencing both the efficacy and safety profile of neuromodulation during prolonged missions.Another promising non-invasive strategy is transcranial direct current stimulation (tDCS), which delivers weak electrical currents to modulate cortical excitability and functional connectivity in targeted brain regions (Polanía et al., 2011). By acting on neural circuits involved in attention, executive function, emotional regulation, and pain processing, tDCS may help counteract disturbances associated with prolonged spaceflight (Romanella et al., 2020). Depending on electrode configuration and stimulation parameters, tDCS could be used to support cognitive performance during high-demand operational periods, reduce maladaptive pain-related processing, or complement sleep- and stress-focused interventions by modulating higher-order cortical control of autonomic and behavioral responses. When integrated into closed-loop systems, tDCS protocols could be personalized based on ongoing physiological and behavioral markers, enabling adaptive stimulation strategies tailored to the astronaut’s current functional state. Such adaptive frameworks will require longitudinal preflight and in-flight characterization of individualized physiological dynamics to distinguish adaptive network remodeling from early maladaptive destabilization, and to define validated thresholds for safely triggering closed-loop interventions during prolonged missions.

Integrating non-pharmacological interventions into AI-guided closed-loop frameworks may represent a key advance for long-duration spaceflight. These systems may detect early deviations from adaptive network states and deliver targeted interventions before apparent dysfunction emerges. Rather than merely suppressing symptoms, the goal is to stabilize the underlying dynamics of interconnected physiological systems and preserve resilience under prolonged stress. For space applications, such technologies must be small, low-power, deployable with minimal reliance on ground-based communication, and interpretable by crew members. Within modulation strategies, radiation-aware constraints could be embedded into closed-loop control, guiding adaptive interventions that support recovery while accounting for mission-specific risk. Neuromodulation protocols should account for potential radiation-induced alterations in neural excitability and vascular integrity to ensure both safety and efficacy across prolonged missions.

## Translating spaceflight research into health on earth

Extreme environments such as space missions and other isolated or confined environments provide a unique opportunity to study how interacting stressors affect human health. In these contexts, individuals face many challenges that affect biological, cognitive, emotional, behavioral, and social functioning. On Earth, such multisystem interactions are difficult to study systematically, as they typically require large populations, long observation periods, and substantial logistical resources (Van Cutsem et al., 2022). Spaceflight and space-analog environments offer a complementary model by enabling longitudinal measurements of physiological and behavioral responses in relatively small cohorts over shorter timeframes (Hardy et al., 2025). These environments can be understood as accelerated models of multisystem stress, where interactions among sleep, stress regulation, pain, cognition, mood, and social dynamics can be continuously tracked. This systems-level perspective allows for the identification of early deviations from adaptive states and the characterization of how dysfunction propagates across interconnected domains. This approach has direct translational relevance for health on Earth. Many common conditions, including chronic pain (Chapman et al., 2008), sleep disorders (Kim et al., 2022), stress-related dysfunction (Ghasemi et al., 2024), and chronic mental fatigue (Pessiglione et al., 2025), are increasingly recognized as emerging from dysregulation across interacting systems. By studying these processes in controlled extreme environments, it may be possible to identify shared mechanisms and intervention targets that are difficult to isolate in more heterogeneous terrestrial populations. This framework may therefore help inform future monitoring and non-pharmacological intervention strategies for multisystem dysfunction on Earth, including controlled isolation facilities, underwater habitats, and Antarctic research stations.

## Conclusion

Long-duration space missions expose astronauts to multiple interacting stressors—including microgravity, ionising radiation, isolation, confinement, and operational demands—that can disrupt interconnected systems involved in sleep, stress regulation, pain processing, cognition, and social interaction. Conceptualizing these processes within a network physiology framework highlight how disturbances may propagate across biological, cognitive, psychological, behavioral, and social domains, thereby increasing the risk of maladaptive outcomes. Advances in sensing and AI-based analysis now enable continuous monitoring of these interactions and the identification of early markers of multisystem alterations. When integrated with non-pharmacological interventions, AI-guided closed-loop systems may provide autonomous, personalized countermeasures to help maintain physiological balance and protect astronaut health during future exploration missions.

## References

[B1] Alzaabi A. A. Alzaabi F. M. Siddiqui R. W. Siddiqui T. W. Sami M. M. (2025). Review of microgravity’s impact on cardiovascular and nervous systems in space exploration. NPJ Microgravity 11, 11. 10.1038/s41526-025-00534-4 41168174 PMC12575774

[B2] Arendt-Nielsen L. Egsgaard L. L. Petersen K. K. Eskehave T. N. Graven-Nielsen T. Hoeck H. C. (2015). A mechanism-based pain sensitivity index to characterize knee osteoarthritis patients with different disease stages and pain levels. Eur. J. Pain 19, 1406–1417. 10.1002/ejp.651 25545011

[B6] Bashan A. Bartsch R. P. Kantelhardt J. W. Havlin S. Ivanov P. C. (2012). Network physiology reveals relations between network topology and physiological function. Nat. Commun. 3, 3. 10.1038/ncomms1705 22426223 PMC3518900

[B7] Blaber A. P. Bondar R. L. Kassam M. S. (2004). Heart rate variability and short duration spaceflight: relationship to post-flight orthostatic intolerance. BMC Physiol. 4, 6. 10.1186/1472-6793-4-6 15113425 PMC420472

[B8] Bu Y. Liang A. Hoffman B. U. Schiehser D. M. Case O. Simmons A. (2026). A review of vagus nerve stimulation for disease: comprehensive theory and evidence for mechanisms of action. Compr. Physiol. 16, e70109. 10.1002/cph4.70109 41781173 PMC12960021

[B9] Capri M. Montano N. Piccirillo S. Narici M. Ferranti F. Maccarrone M. (2025). Spaceflight exposome/microgravity effects on the psychoimmunoneuroendocrine system. NPJ Microgravity 11, 11. 10.1038/s41526-025-00487-8 41006313 PMC12475253

[B10] Ceniza-Bordallo G. Zimmermann E. Vigouroux M. Niburski K. Fortin M. Ouellet J. (2024). Low back pain during and after spaceflight: a systematic review with meta-analysis. J. Pain Res. 17, 4103–4139. 10.2147/JPR.S491060 39660277 PMC11630706

[B11] Chapman C. R. Tuckett R. P. Song C. W. (2008). Pain and stress in a systems perspective: reciprocal neural, endocrine and immune interactions. J. Pain 9, 122–145. 10.1016/j.jpain.2007.09.006 18088561 PMC2278005

[B12] Chunlai L. Chi W. Yong W. Yangting L. (2019). China’s present and future lunar exploration program. Science 365, 238–239. 10.1126/science.aax9908 31320531

[B13] Demontis G. C. Germani M. M. Caiani E. G. Barravecchia I. Passino C. Angeloni D. (2017). Human pathophysiological adaptations to the space environment. Front. Physiol. 8, 1–17. 10.3389/fphys.2017.00547 28824446 PMC5539130

[B14] Etemadi M. Inan O. T. (2018). Emerging wearable physiological monitoring technologies and decision aids for health and performance. J. Appl. Physiol. 124, 452–461. 10.1152/japplphysiol.00298.2017 29097633

[B15] Friedman E. Bui B. (2017). A psychiatric formulary for long-duration spaceflight. Aerosp. Med. Hum. Perform. 88, 1024–1033. 10.3357/AMHP.4901.2017 29046178

[B16] Gao W. Emaminejad S. Nyein H. Y. Y. Challa S. Chen K. Peck A. (2016). Fully integrated wearable sensor arrays for multiplexed *in situ* perspiration analysis. Nature 529, 509–514. 10.1038/nature16521 26819044 PMC4996079

[B17] Gerhart J. I. Burns J. W. Post K. M. Smith D. A. Porter L. S. Burgess H. J. (2018). Relationships between sleep quality and pain-related factors for people with chronic low back pain: tests of reciprocal and time of day effects. Ann. Behav. Med. 51, 365–375. 10.1007/s12160-016-9860-2 27844327 PMC5846493

[B18] Ghasemi F. Beversdorf D. Q. Herman K. C. (2024). Stress and stress responses: a narrative literature review from physiological mechanisms to intervention approaches. J. Pac Rim Psychol. 18, 18. 10.1177/18344909241289222

[B19] Ghosh S. Kim S. Ijaz M. F. Singh P. K. Mahmud M. (2022). Classification of mental stress from wearable physiological sensors using image-encoding-based deep neural network. Biosensors 12, 1153. 10.3390/bios12121153 36551120 PMC9775098

[B20] Gkikas S. Tsiknakis M. (2023). Automatic assessment of pain based on deep learning methods: a systematic review. Comput. Methods Programs Biomed. 231, 107365. 10.1016/j.cmpb.2023.107365 36764062

[B21] Grigor’ev A. I. Orlov O. I. Baranov V. M. (2021). Space medicine: scientific foundations, achievements, and challenges. Her. Russ. Acad. Sci. 91, 626–629. 10.1134/S1019331621060022 35125838 PMC8807369

[B22] Hardy J. G. Theriot C. A. Oswald T. Clément G. (2025). Spaceflight standard measures is a multidisciplinary study that systematically monitors risks to astronaut health and performance. NPJ Microgravity 11, 11. 10.1038/s41526-025-00532-6 41224762 PMC12612093

[B23] Hassanpour A. Yang B. (2025). Contactless vital sign monitoring: a review towards multi-modal multi-task approaches. Sensors 25, 25. 10.3390/s25154792 PMC12349365 40807957

[B24] Heikenfeld J. Jajack A. Rogers J. Gutruf P. Tian L. Pan T. (2018). Wearable sensors: modalities, challenges, and prospects. Lab. Chip 18, 217–248. 10.1039/c7lc00914c 29182185 PMC5771841

[B25] Herman J. P. McKlveen J. M. Ghosal S. Kopp B. Wulsin A. Makinson R. (2016). Regulation of the hypothalamic-pituitary-adrenocortical stress response. Compr. Physiol. 6, 603–621. 10.1002/cphy.c150015 27065163 PMC4867107

[B26] Hirvonen J. Monto S. Wang S. H. Palva J. M. Palva S. (2018). Dynamic large-scale network synchronization from perception to action. Netw. Neurosci. 2, 442–463. 10.1162/netn_a_00039 30320293 PMC6175692

[B27] Jones C. W. Basner M. Mollicone D. J. Mott C. M. Dinges D. F. (2022). Sleep deficiency in spaceflight is associated with degraded neurobehavioral functions and elevated stress in astronauts on six-month missions aboard the international space station. Sleep 45, 45. 10.1093/sleep/zsac006 PMC8919197 35023565

[B28] Kim H. G. Cheon E. J. Bai D. S. Lee Y. H. Koo B. H. (2018). Stress and heart rate variability: a meta-analysis and review of the literature. Psychiatry Investig. 15 (08.17), 235–245. 10.30773/pi.2017 PMC5900369 29486547

[B29] Kim H. Jung H. R. Kim J. B. Kim D. J. (2022). Autonomic dysfunction in sleep disorders: from neurobiological basis to potential therapeutic approaches. J. Clin. Neurol. 18, 140–151. 10.3988/jcn.2022.18.2.140 35274834 PMC8926769

[B30] Kuldavletova O. Navarro Morales D. C. Quarck G. Denise P. Clément G. (2023). Spaceflight alters reaction time and duration judgment of astronauts. Front. Physiol. 14, 14. 10.3389/fphys.2023.1141078 PMC10063900 37007995

[B31] Lane N. D. Bhattacharya S. Georgiev P. Forlivesi C. Jiao L. Qendro L. (2016). “DeepX: a software accelerator for low-power deep learning inference on Mobile devices,” in Proceedings of the 15th ACM/IEEE International Conference on Information Processing in Sensor Networks (IPSN) (Vienna: ACM), 1415–1426.

[B32] Lazarus R. S. (1993). From psychological stress to the emotions: a history of changing outlooks. Annu. Rev. Psychol. 44, 1–21. 10.1146/annurev.ps.44.020193.000245 8434890

[B33] Lee A. G. Mader T. H. Gibson C. R. Tarver W. Rabiei P. Riascos R. F. (2020). Spaceflight associated neuro-ocular syndrome (SANS) and the neuro-ophthalmologic effects of microgravity: a review and an update. NPJ Microgravity 6, 6. 10.1038/s41526-020-0097-9 32047839 PMC7005826

[B34] Lee P. H. U. Chung M. Ren Z. Mair D. B. Kim D. H. (2022). Factors mediating spaceflight-induced skeletal muscle atrophy. Am. J. Physiol. Cell Physiol. 322, C567–C580. 10.1152/ajpcell.00203.2021 35171699

[B35] Li F. Zhang D. (2025). Multimodal physiological signals from wearable sensors for affective computing: a systematic review. Intell. Sports Health 1, 210–222. 10.1016/j.isph.2025.04.001

[B36] Lo Martire V. Caruso D. Palagini L. Zoccoli G. Bastianini S. (2020). Stress & sleep: a relationship lasting a lifetime. Neurosci. Biobehav Rev. 117, 65–77. 10.1016/j.neubiorev.2019.08.024 31491473

[B37] Macho L. Koska J. Ksinantova L. Pacak K. Hoff T. Noskov V. B. (2003). The response of endocrine system to stress loads during space flight in human subject. Adv. Space Res. 31, 1605–1610. 10.1016/s0273-1177(03)00097-8 12971416

[B38] Maher C. Underwood M. Buchbinder R. (2017). Non-specific low back pain. Lancet 389, 736–747. 10.1016/S0140-6736(16)30970-9 27745712

[B39] Man J. Graham T. Squires-Donelly G. Laslett A. L. (2022). The effects of microgravity on bone structure and function. NPJ Microgravity 8, 8. 10.1038/s41526-022-00194-8 35383182 PMC8983659

[B40] Mariotti A. (2015). The effects of chronic stress on health: new insights into the molecular mechanisms of brain-body communication. Future Sci. OA 1, 1. 10.4155/fso.15.21 28031896 PMC5137920

[B41] Mason C. E. Green J. Adamopoulos K. I. Afshin E. E. Baechle J. J. Basner M. (2024). A second space age spanning omics, platforms and medicine across orbits. Nature 632, 995–1008. 10.1038/s41586-024-07586-8 38862027 PMC12366838

[B4] Mohamed D. Philippe R. Olivier B. (2018). Use of cardiorespiratory coherence to separate spectral bands of the heart rate variability. Biomed. Signal Process Control. 10.1016/j.bspc.2018.08.003

[B5] Mohsen N. Giri P. K. Elizabeth A. M. (2019). Coupling of autonomic and central events during sleep benefits declarative memory consolidation. Neurobiol. Learn Mem. 157:139–150. 10.1016/j.nlm.2018.12.008 30562589 PMC6425961

[B42] Moor M. Banerjee O. Abad Z. S. H. Krumholz H. M. Leskovec J. Topol E. J. (2023). Foundation models for generalist medical artificial intelligence. Nature 616, 259–265. 10.1038/s41586-023-05881-4 37045921

[B43] Morokuma S. Hayashi T. Kanegae M. Mizukami Y. Asano S. Kimura I. (2023). Deep learning-based sleep stage classification with cardiorespiratory and body movement activities in individuals with suspected sleep disorders. Sci. Rep. 13, 17730. 10.1038/s41598-023-44785-3 37853134 PMC10584883

[B44] Mukkamala R. Hahn J. O. Inan O. T. Mestha L. K. Kim C. S. Toreyin H. (2015). Toward ubiquitous blood pressure monitoring via pulse transit time: theory and practice. IEEE Trans. Biomed. Eng. 62, 1879–1901. 10.1109/TBME.2015.2441951 26057530 PMC4515215

[B45] Myers B. McKlveen J. M. Herman J. P. (2014). Glucocorticoid actions on synapses, circuits, and behavior: implications for the energetics of stress. Front. Neuroendocrinol. 35, 180–196. 10.1016/j.yfrne.2013.12.003 24361584 PMC4422101

[B46] NASA (2020). Nasa’s Lunar Exploration Program Overview. Available online at: https://www.nasa.gov/sites/default/files/atoms/files/artemis_plan-20200921.pdf (Accessed September 21, 2020).

[B47] Pagnini F. Phillips D. Bercovitz K. Langer E. (2019). Mindfulness and relaxation training for long duration spaceflight: evidences from analog environments and military settings. Acta Astronaut. 165, 1–8. 10.1016/j.actaastro.2019.07.036

[B48] Park S. A. Lee H. C. Jung C. W. Yang H. L. (2022). “Attention mechanisms for physiological signal deep learning: which attention should we take?,” in Medical Image Computing and Computer Assisted Intervention – MICCAI 2022. Editors Wang L. Dou Q. Fletcher P. T. Speidel S. Li S. (Cham: Springer Nature Switzerland), 613–622.

[B49] Pessiglione M. Blain B. Wiehler A. Naik S. (2025). Origins and consequences of cognitive fatigue. Trends Cogn. Sci. 29, 730–749. 10.1016/j.tics.2025.02.005 40169294

[B50] Polanía R. Nitsche M. A. Paulus W. (2011). Modulating functional connectivity patterns and topological functional organization of the human brain with transcranial direct current stimulation. Hum. Brain Mapp. 32, 1236–1249. 10.1002/hbm.21104 20607750 PMC6870160

[B51] Raja S. N. Carr D. B. Cohen M. Finnerup N. B. Flor H. Gibson S. (2020). The revised IASP definition of pain: concepts, challenges, and compromises. Pain 161, 1976–1982. 10.1097/j.pain.0000000000001939 32694387 PMC7680716

[B52] Romanella S. M. Sprugnoli G. Ruffini G. Seyedmadani K. Rossi S. Santarnecchi E. (2020). Noninvasive brain stimulation and space exploration: opportunities and challenges. Neurosci. Biobehav Rev. 119, 294–319. 10.1016/j.neubiorev.2020.09.005 32937115 PMC8361862

[B53] Scott J. Feiveson A. H. English K. L. Spector E. R. Sibonga J. D. Lichar D. E. (2023). Effects of exercise countermeasures on multisystem function in long duration spaceflight astronauts. NPJ Microgravity 9, 9. 10.1038/s41526-023-00256-5 36737441 PMC9898566

[B54] Scully R. R. Basner M. Nasrini J. Lam C. W. Hermosillo E. Gur R. C. (2019). Effects of acute exposures to carbon dioxide on decision making and cognition in astronaut-like subjects. NPJ Microgravity 5, 5. 10.1038/s41526-019-0071-6 31240239 PMC6584569

[B55] Shanthanna H. Nelson A. M. Kissoon N. Narouze S. (2022). The COVID-19 pandemic and its consequences for chronic pain: a narrative review. Anaesthesia 77, 1039–1050. 10.1111/anae.15801 35848380 PMC9350079

[B56] Shields G. S. Sazma M. A. Yonelinas A. P. (2016). The effects of acute stress on core executive functions: a meta-analysis and comparison with cortisol. Neurosci. Biobehav Rev. 68, 651–668. 10.1016/j.neubiorev.2016.06.038 27371161 PMC5003767

[B57] Singh S. Beuria J. Behera L. Bilas Pachori R. Gupta V. (2026). Decoding cognitive load changes induced by mantra meditation from physiological signals using deep neural networks. IEEE Sens. J. 26, 1088–1102. 10.1109/JSEN.2025.3531942

[B58] Tays G. D. Hupfeld K. E. McGregor H. R. Salazar A. P. De Dios Y. E. Beltran N. E. (2021). The effects of long duration spaceflight on sensorimotor control and cognition. Front. Neural Circuits 15, 15. 10.3389/fncir.2021.723504 34764856 PMC8577506

[B3] Terry B. J. Chun-Yu C. Ya-Chuan H. (2016). EEG beta power and heart rate variability describe the association between cortical and autonomic arousals across sleep. Auton. Neurosci. 10.1016/j.autneu.2015.12.001 26681575

[B59] Thapa R. Kjaer M. R. He B. Covert I. Moore H. Hanif U. (2026). A multimodal sleep foundation model for disease prediction. Nat. Med., 1–11. 10.1038/s41591-025-03487-1 41495409 PMC12920147

[B60] Van Cutsem J. Abeln V. Schneider S. Keller N. Diaz-Artiles A. Ramallo M. A. (2022). The impact of the COVID-19 lockdown on human psychology and physical activity; A space analogue research perspective. Int. J. Astrobiol. 21, 32–45. 10.1017/S1473550421000409

[B61] van Oosterhout W. P. J. Perenboom M. J. L. Terwindt G. M. Ferrari M. D. Vein A. A. (2024). Frequency and clinical features of space headache experienced by astronauts during long-haul space flights. Neurology 102, e209224. 10.1212/WNL.0000000000209224 38478846 PMC11033988

[B62] Vinokhodova A. Gushin V. Kuznetsova P. Yusupova A. (2023). Crew interaction in extended space missions. Aerospace 10, 10. 10.3390/aerospace10080719

[B63] Willey J. S. Britten R. A. Blaber E. Tahimic C. G. T. Chancellor J. Mortreux M. (2021). The individual and combined effects of spaceflight radiation and microgravity on biologic systems and functional outcomes. J. Environ. Sci. Health C Toxicol. Carcinog. 39, 129–179. 10.1080/26896583.2021.1885283 33902391 PMC8274610

[B64] Wotring V. E. (2015). Medication use by U.S. crewmembers on the international space station. FASEB J. 29, 4417–4423. 10.1096/fj.14-264838 26187345

[B65] Yap Y. Slavish D. C. Taylor D. J. Bei B. Wiley J. F. (2020). Bi-directional relations between stress and self-reported and actigraphy-assessed sleep: a daily intensive longitudinal study. Sleep 43, 43. 10.1093/SLEEP/ZSZ250 31608395 PMC7066487

[B66] Yildiz O. Subasi A. (2026). PAMG-AT: a physiological attention multi-graph model with adaptive topology for stress detection using wearable devices. bioRxiv. 10.1101/2026.03

[B67] Yin Y. Liu J. Fan Q. Zhao S. Wu X. Wang J. (2023). Long-term spaceflight composite stress induces depression and cognitive impairment in astronauts—insights from neuroplasticity. Transl. Psychiatry 13, 13. 10.1038/s41398-023-02638-5 37938258 PMC10632511

[B68] Zea L. Warren L. Ruttley T. Mosher T. Kelsey L. Wagner E. (2024). Orbital reef and commercial low Earth orbit destinations—upcoming space research opportunities. NPJ Microgravity 10, 43. 10.1038/s41526-024-00363-x 38553503 PMC10980796

[B69] Zong H. Fei Y. Liu N. (2025). Circadian disruption and sleep disorders in astronauts: a review of multi-disciplinary interventions for long-duration space missions. Int. J. Mol. Sci. 26, 26. 10.3390/ijms26115179 40507991 PMC12155433

[B70] Zrenner C. Ziemann U. (2024). Closed-Loop brain stimulation. Biol. Psychiatry 95, 545–552. 10.1016/j.biopsych.2023.09.014 37743002 PMC10881194

